# Concurrent Neoadjuvant Chemotherapy and Radiation in Locally Advanced Breast Cancer: Impact on Locoregional Recurrence Rates

**DOI:** 10.3390/curroncol32020085

**Published:** 2025-02-01

**Authors:** Natalie Grindrod, Matthew Cecchini, Muriel Brackstone

**Affiliations:** 1 Schulich Faculty of Medicine & Dentistry, Western University, London, ON N6A 3K7, Canada; natalie.grindrod@lhsc.on.ca (N.G.); matthew.cecchini@lhsc.on.ca (M.C.); 2 Department of Pathology, London Health Sciences Centre, London, ON N6A 5A5, Canada; 3 Department of Surgery, London Health Sciences Centre, London, ON N6A 5W9, Canada

**Keywords:** breast cancer, chemotherapy, radiation, tumor-infiltrating lymphocytes, complete response, recurrence

## Abstract

Neoadjuvant chemoradiation therapy (NCRT) is an underutilized treatment in breast cancer but may improve outcomes by impacting the tumor immune microenvironment. The aim of this study was to evaluate NCRT’s impact on recurrence and the role of tumor-infiltrating lymphocytes (TILs) in treatment response. We hypothesized that NCRT reduces recurrence by upregulating TILs. Patients with locally advanced breast cancer (LABC) were treated with NCRT. Stage IIB to III patients with any molecular subtypes were eligible. The patients were matched for age, stage, and molecular subtype by a propensity score to a concurrent cohort receiving standard neoadjuvant chemotherapy (NCT) followed by adjuvant radiation. The objective of this study was to assess the patients in terms of the pathological complete response (pCR), TIL counts prior to and following treatment, and locoregional recurrence. The median follow-up was 7.2 years. Thirty NCRT patients were successfully matched 1:3 to ninety NCT patients. The NCRT cohort had no regional and locoregional recurrences (*p* = 0.036, (hazard ratio) HR [0.25], 95% confidence interval (CI) [0.06–0.94] and *p* = 0.013, HR [0.25], 95% CI [0.08–0.76], respectively), compared to 17.8% of the NCT cohort. The NCRT group had significantly more pCRs, and TILs were increased in the post-treatment pCR specimens. NCRT can improve outcomes in LABC patients, with a higher pCR and significantly lower locoregional recurrence/higher recurrence-free survival. Further trials are needed to evaluate the role of NCRT in all breast cancer patients.

## 1. Introduction

Breast cancer is the most common cancer among women in Canada, with over 29,000 diagnosed in 2023 [1]. Locally advanced breast cancer (LABC) is defined as Stages IIB and III [2,3]. As these cancers tend to be larger or with more extensive nodal involvement, neoadjuvant chemotherapy (NCT) is considered an appropriate clinical standard to cytoreduce disease [2,4,5]. The majority of patients are able to down-stage their disease with neoadjuvant treatment, which can then make them eligible for breast-conserving surgery [4,5]. Neoadjuvant treatment can also result in a pathological complete response (pCR), meaning no residual disease in the breast or axilla at the time of surgery, providing a powerful prognostic surrogate for a favorable long-term outcome and survival [2]. Additionally, the response to NCT provides important information for clinicians regarding the survival benefit with the addition of adjuvant systemic treatments based on subtype (Kadcyla for Her2+ patients with residual disease [6]; CDK4/6 inhibitor for ER+ disease [7], and capecitabine for triple-negative disease [8]). The findings of pCR after neoadjuvant treatment can be used to de-escalate therapy; for example, axillary radiation can be avoided in patients achieving pCR after NCT [9]. Additionally, breast-conserving surgery can be offered pending a sufficient reduction in the tumor bulk, and sentinel node biopsy can safely replace axillary dissection in those who have been rendered clinically node-negative after NCT [10].

Breast tumor biomarkers, such as tumor-infiltrating lymphocytes (TILs), have been evaluated to determine how they may play a role in providing information to tailor patient treatment based on their response to neoadjuvant treatment [11]. TILs are present in tumors exhibiting an immune response either within the tumor and/or the surrounding stroma [12,13]. Since radiation can be used to kill tumor cells by double-stranded DNA damage, the tumor antigens released during radiation may also sensitize immune cells [12] and may explain historic data suggesting a survival benefit with radiation when delivered in the neoadjuvant setting [14]. Further, cancer cells may express programmed death (PD) ligand 1 (PD-L1), which can communicate with PD-1 receptors on immune cells such as TILs, in order to evade immune system-mediated cell death [13,15,16,17,18].

Published research supports the utilization of TILs as prognostic and predictive markers in patients treated with chemotherapy and immunotherapy [13]. High TILs were found to correlate with pCR, a surrogate for overall survival. Among LABC patients treated with NCT (+/− immunotherapy), those achieving pCR correlated with high TILs at baseline [19,20,21,22]. To date, only one study has evaluated neoadjuvant radiation (NRT), suggesting that radiation therapy (RT) may increase stromal TILs (sTILs) [23].

To increase the understanding of the impact of RT on TILs and to explore whether neoadjuvant chemoradiation therapy (NCRT) increases pCR and recurrence-free survival by upregulating TILs, archived tumor slides were evaluated from a locally conducted Phase II clinical trial of NCRT vs. NRT in patients with advanced breast cancer [24]. Our hypothesis was that NCRT would improve clinical outcomes in LABC patients by upregulating TILs.

## 2. Methods

**Figure 1:**
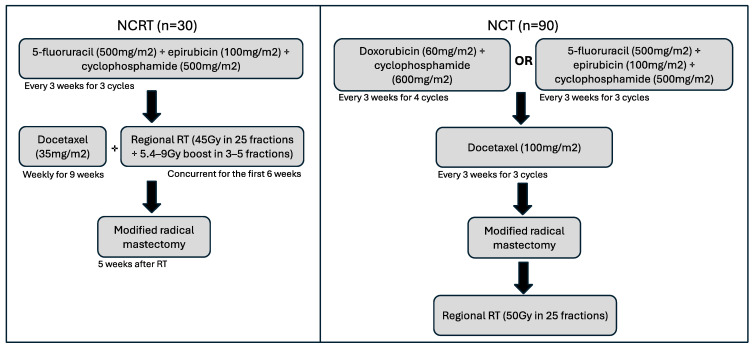
Schematic of treatment timelines and cycles for both NCRT and NCT cohorts. Following these regimens, patients received standard-of-care treatments, including endocrine therapy for those with hormone receptor-positive disease or trastuzumab for HER2-positive breast cancers.

A previous Phase II single-arm clinical trial enrolled patients with newly diagnosed Stage IIB/III invasive breast cancer who were treated with NCRT (n = 32) [24]. These study patients received 5-fluorouracil (500 mg/m^2^), epirubicin (100 mg/m^2^), and cyclophosphamide (500 mg/m^2^) (FEC) every 3 weeks, for 3 cycles, followed by weekly docetaxel (35 mg/m^2^) for a total of 9 weeks (Figure 1). Regional RT (45 Gy in 25 fractions with 5.4–9 Gy boost in 3–5 fractions) was delivered concurrently for the first 6 weeks of docetaxel. Then, patients went on to receive modified radical mastectomy 5 weeks following the completion of RT. In addition, patients with HER2-positive disease were initiated on trastuzumab every 3 weeks at the time of starting docetaxel, for 1 year (18 doses). Patients with estrogen receptor (ER)-positive disease were given adjuvant endocrine therapy in accordance with clinician preference and patient menopausal status.

Patients in the control cohort of NCT (n = 90) underwent standard care at the time of study with either doxorubicin (60 mg/m^2^) and cyclophosphamide (600 mg/m^2^) every 3 weeks for 4 cycles followed by docetaxel (100 mg/m^2^) every 3 weeks for 3 cycles, or 5-fluorouracil (500 mg/m^2^), epirubicin (100 mg/m^2^), and cyclophosphamide (500 mg/m^2^) every 3 weeks for 3 cycles, then docetaxel (100 mg/m^2^) every 3 weeks for 3 cycles (Figure 1). Patients then underwent a modified radical mastectomy before receiving adjuvant standard RT of 50 Gy in 25 fractions using intensity-modulated RT. Patient outcomes were recorded (pCR and locoregional recurrence).

A sample size of 52 in the single-arm NCRT trial was calculated to be powered to identify a doubling in pCR rate from 13% at the time, to 26% (using 0.05 alpha error and 0.2 beta error rates using two-sided tail, using an online sample size calculator) [25]. The study was terminated early after 32 patients had been treated due to concerns of chemical pneumonitis with docetaxel at the time. Thirty of those patients were successfully matched using propensity score greedy matching (+/−0.1) based on covariates impacting clinical outcome (grade, molecular subtype, and patient age) 3:1 to ninety control patients being treated with standard NCT, surgery, and then adjuvant radiation. Patients were enrolled and treated from 2010 to 2012, with a median follow-up of 7.2 years.

Examining the patients’ pathology slides (both baseline core biopsy and surgical pathology slides) and interpreting TILs by study cohort and clinical outcome was approved by the University of Western Ontario’s (London, ON, Canada) Human Subjects Research Ethics Board Study#105643. All slides underwent image analysis for TIL assessment. Archived tumor blocks were obtained from the pathology department to request a hematoxylin and eosin (H&E) diagnostic biopsy slide and a surgical post-neoadjuvant therapy slide of the tumor bed for each of the 120 study patients. Out of 120 patient pre-treatment biopsy samples, there were 117 cases cut and stained for immunohistochemistry (IHC), as 3 cases had inadequate tissue remaining. These 240 H&E slides were scanned using the Aperio AT slide scanner at 40× magnification. IHC stains included CD3, CD4, CD8, CD45, CD68, CD1a, PAX5, and Myeloperoxidase (MPO). IHC was performed using the Dako Omnis system (version 7, Agilent Technologies, Santa Clara, CA, USA), with the following product codes for the respective stains: GA503, IR649, GA623, GA751, GA609, IR069, IR650, and GA511. Slides had positive and negative controls embedded and expected staining patterns served as an internal control. Laboratory practices were standardized and machines were used when appropriate, minimizing variation.

### 2.1. Image Analysis

QuPath version 0.5, an open-source software, was used for the image analysis. Slides were imported into a QuPath project file and underwent image analysis streamlined with coding for semi-automation. Image analysis included cell detection, object classifiers, and pixel classifiers, utilizing an artificial neural network for the classifying. Tumor, stroma, and immune cells were identified in each case of H&E slides for the object and pixel classifiers. Guidelines established by the International Immuno-Oncology Biomarker Working Group on Breast Cancer were followed [11,26,27,28]. This included enumerating TILs across the selected area of the slide, which excluded areas of artifacts, normal breast tissue, tertiary lymphoid structures, and others defined further in the guidelines. The stromal and intratumoral areas were defined by utilizing the artificial neural network again with a pixel classifier; therefore, the enumeration of TILs could be counted as total, stromal, or intratumoral. Counts of tumor cells and stroma cells were also collected for developing a percentage of TILs as a continuous variable. For the IHC slides, only positive cell detection was performed. 

### 2.2. Data Analysis

Data were collected including tumor, stroma, and immune cell counts for each H&E slide, and percentage of cells positively stained for the respective IHC stains. These were analyzed in R version 4.2.2, a statistical software, utilizing packages ‘dplyr’, ‘ggplot2’, ‘survival’, ‘survminer’, ‘gtsummary’, and ‘kableExtra’, and built-in base packages [29]. Data collected on patients were analyzed for survival and outcomes, and additionally compared to measurements gathered from QuPath. Non-parametric tests were utilized, including Kruskal–Wallis, Chi-squared, and Fisher’s Exact Tests. Kaplan–Meier survival curve plots were used to evaluate local and regional recurrence-free survival, using log rank tests.

## 3. Results

The 120 patients included in this study had varying disease characteristics including the molecular subtype, tumor grade, and stage (Table 1). However, the propensity score matching was based on the age, grade, and molecular subtype, ensuring they were well matched in terms of demographic variables (Table 1). The majority of the patients had an initial grade of 2 or 3, and most had a TNM staging of T3N1M0 (Table 1). All patients were between Stage IIA and Stage IIIC, with most having Stage IIIA breast cancer (Table 1). Most patients were ER- and progesterone receptor (PR)-positive or HER2-negative, while some had triple-negative breast cancer (TNBC) and HER2-positive disease. None of these characteristics were significantly different between treatment groups using Fisher’s exact test and Chi-squared test.

### 3.1. Treatment Response

These patients’ clinical responses to their neoadjuvant treatment were largely categorized as partial responses (103 of 120), with some having a complete response (12), others remaining stable (3), and very few progressing on treatment (2). The NCRT cohort did not have any patients with progressive or stable disease; all responded to treatment.

**Figure 2:**
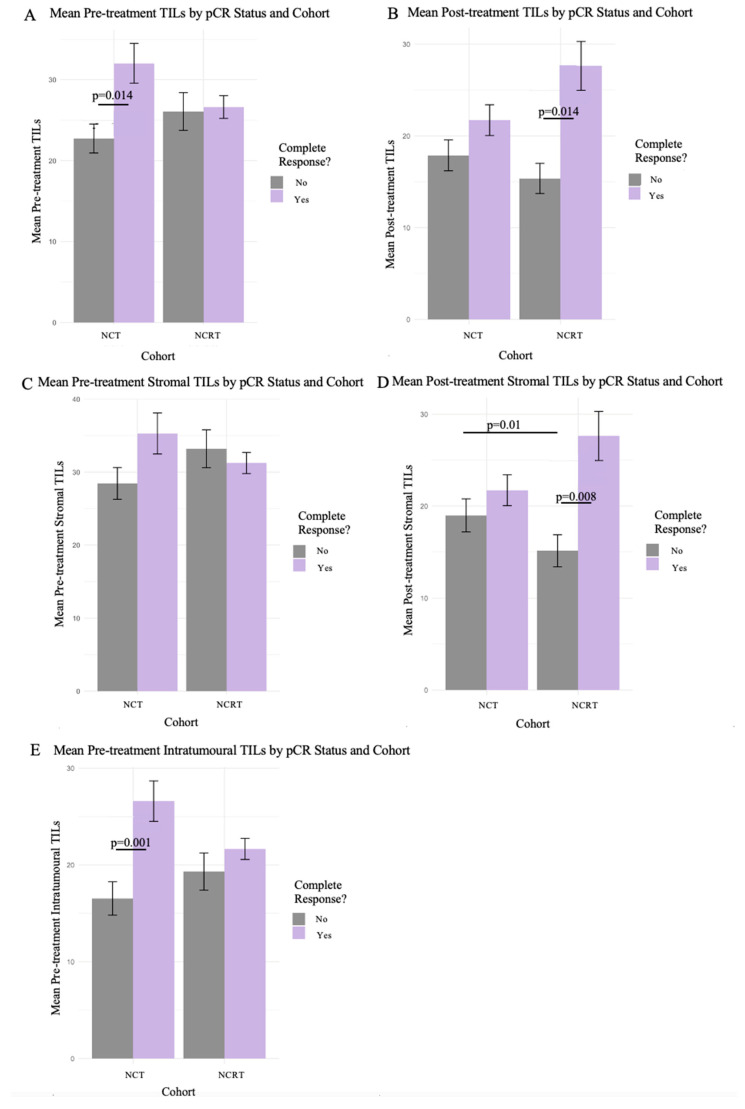
Pathological complete response between cohorts with the various methods of TIL assessments. Mean TIL counts as percentage shown in bar plots with 95% confidence intervals for pathological complete response between cohorts. Includes the 3 different methods of evaluating H&E specimen TILs: total, stromal, and intratumoral. Wilcoxon’s Rank Sum test was used for significance between pCR status bars, and the Kruskal–Wallis test was used for comparison between cohorts. (**A**,**C**,**E**) depict the cohorts with mean total, stromal, and intratumoral TIL percentage for pCR in pre-treatment specimens, respectively. (**B**,**D**) depict the cohorts with mean total and stromal TIL percentage for pCR in post-treatment specimens, respectively.

At surgery, 22 (16%) patients had pCR, with 7 (23%) belonging to the NCRT cohort (*p* < 0.001, Phi coefficient = 0.07), as previously reported [24,30]. Patients achieving pCR tended to have a higher percentage of TILs in both the pre-treatment and post-treatment specimens (Figure 2). The patients in the NCT cohort who achieved pCR had a significantly higher TIL count in the pre-treatment specimens (Figure 2A,E), while patients in the NCRT cohort who achieved pCR had a significantly higher TIL count in the post-treatment specimens (Figure 2B,D). The IHC analysis of CD3, CD4, CD8, CD45, CD68, CD1a, PAX5, and MPO stains were exploratory and did not appear to differ in response to the treatment or treatment cohort and were therefore not included in this analysis.

### 3.2. Outcomes

**Figure 3:**
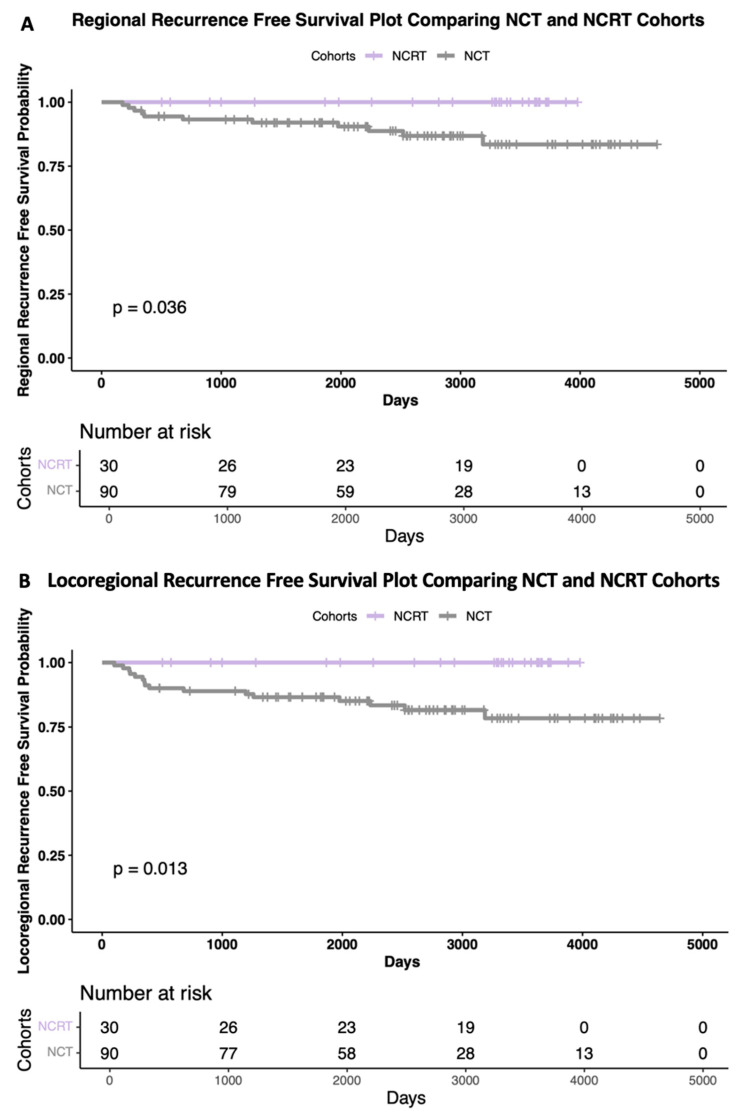
Kaplan–Meier survival curves. Regional (**A**), and locoregional (**B**) recurrence-free survival plots comparing chemotherapy (grey) and chemoradiotherapy (purple) cohorts. Risk tables included below each plot. Log rank tests were used for significance tests.

Overall, 38 out of 120 patients had recurrences. Eight distant recurrences occurred in the NCRT cohort (26.7%) and thirty local, regional, and distant recurrences occurred in the NCT cohort (33.3%). None of the NCRT cohort had a local or regional recurrence, but 16 patients in the NCT cohort did. The Kaplan–Meier survival curves are shown in Figure 3, comparing the cohorts for regional (A) and locoregional (B) recurrences, with both having significant differences, as evidenced by the significantly lower recurrences in the NCRT cohort (*p* = 0.036, hazard ratio (HR) [0.25], 95% confidence interval (CI) [0.06–0.94] and *p* = 0.013, HR [0.25], 95% CI [0.08–0.76], respectively).

## 4. Discussion

This study’s previous reports [24,30] demonstrated that the NCRT cohort achieved a significantly higher rate of pathological complete responses than the NCT cohort with early follow-up. At that time, there had been no significance seen in the overall survival, recurrence-free survival, or disease-free survival. With longer follow-up, we are now able to demonstrate a significant difference between the treatment cohorts for regional and locoregional recurrence-free survival. Figure 3 illustrates the Kaplan–Meier curves, where the NCRT cohort had zero local and zero regional recurrences, unlike the NCT cohort. The findings of the significantly higher pCR rates with NCRT [24,30] are in keeping with the Stockholm trial of NRT in breast cancer, which demonstrated significant improvements in survival and reduced distant recurrences for patients treated with NRT compared to those receiving adjuvant RT or no RT [31]. These findings support the hypothesis that NRT delivered to a tumor in situ may prime the immune system and that NRT may have a greater role in breast cancer than its current use, which is for improving the resectability of inoperable cancers [32,33,34,35]. In another study comparing adjuvant to NRT, the NRT cohort had significantly fewer patients who developed a second primary breast cancer, likely due to radiation-induced antitumor immunity-based effects [33]. NRT should be investigated further to determine the most beneficial dosage and fractionation and should also be considered in the treatment of both early and advanced breast cancer.

This study explored the applicability of utilizing digital pathology to improve the assessment of TILs. There was not any predictive or prognostic effect of TILs in terms of survival, despite prior studies demonstrating that TILs are predictive of chemotherapeutic outcomes [13]. The baseline TILs in the chemotherapy cohort were predictive of pCR, where the patients with higher TILs were more likely to achieve a pathological complete response (Figure 2A,E). This supports the existing literature suggesting that higher baseline TILs may predict pCR [13]. Additionally, the NCRT cohort had significantly more TILs in those who achieved a pCR. This aligns with our hypothesis that NRT induces immune-mediated protection against breast cancer recurrence [12], which is further evidenced by the only other TIL study with NRT, which demonstrated that radiotherapy could increase TILs and MHC-I expression [23]. This study also supports findings from the early work in the field of TILs in LABC patient cohorts [20,22,36,37] for all molecular subtypes. Our study included all molecular subtypes given that it was exploratory and designed to examine which molecular subtypes may benefit from NCRT. As such, this heterogeneity may have impacted a lack of statistical power in our existing sample size. More clinical trials are needed to better understand how to use TILs most effectively and which specific patient cohorts this biomarker should be utilized for.

NRT may improve survival, local control, and resectability not only through its ability to kill replicating cancer cells by double-stranded DNA damage, but also through potentially inducing a sustained immune response [12], reducing the recurrence risk by avoiding immunotolerance of cancer recurrence. The dosage and fractionation used in this LABC Chemoradiation Trial are not the hypofractionated regimens that have more recently been proposed for optimal immune modulation; therefore, the impact on recurrence or survival may be greater with hypofractionated regimens. Combining NRT with an immune checkpoint inhibitor (ICI) that targets PD-1 receptors could allow for the priming of optimal immune responses against breast cancer recurrence. Further studies exploring the use of NRT with immunotherapy are ongoing, and these findings need to be validated in larger prospective randomized controlled trials utilizing a hypofractionated radiation treatment regimen [3]. A limitation of this study is that the sample size was small, specifically for the NCRT cohort, limiting the strength of the conclusions. Having said that, the study was sufficiently powered to detect a significant difference in the recurrence-free survival. This work should be replicated with current hypofractionated regimens and a larger sample size to validate these findings. Further, there was no randomization to a control arm to bias as the study was conducted as a prospective single-arm intervention. The risk of bias was addressed using a blinded statistician to perform propensity score matching for covariates known to impact recurrence. However, there always remains a risk in matched analysis that additional factors may bias these findings. The power of this study was to detect a significant difference in pCR, which it successfully demonstrated. There was no sample size calculation for the secondary and exploratory analysis of the TILs. It is possible that the differences in the TILs are correlated to outcomes in both study arms but that we were unable to detect them given this study’s small sample size. We hope to repeat this analysis in a larger prospective randomized trial in order to further evaluate the role of TILs in chemoradiation for breast cancer.

## 5. Conclusions

Neoadjuvant chemotherapy with concurrent neoadjuvant radiation significantly improved pCR and recurrence-free survival in patients with advanced-stage breast cancer, with none of the patients treated in the neoadjuvant radiation cohort experiencing locoregional recurrence. The potential role of neoadjuvant radiation in immune priming against cancer recurrence needs to be better understood and clinicians should consider a paradigm shift where there may be greater benefit in changing current treatment algorithms to provide radiation in the neoadjuvant setting for all breast cancer patients.

## Figures and Tables

**Table 1:** Disease characteristics of dataset.

Variable	N	NCT, N = 90 ^1^	NCRT, N = 30 ^1^	*p*-Value ^2^
**Biopsy Grade**	120			0.7
1		5 (5.6%)	2 (6.7%)	
2		39 (43%)	15 (50%)	
3		46 (51%)	13 (43%)	
**ER Result**	120			0.7
Negative		24 (27%)	7 (23%)	
Positive		66 (73%)	23 (77%)	
**PR Result**	120			0.7
Negative		30 (33%)	11 (37%)	
Positive		60 (67%)	19 (63%)	
**HER2 Status**	120			>0.9
Negative		61 (68%)	20 (67%)	
Positive		29 (32%)	10 (33%)	
**Clinical T**	120			0.4
T1		1 (1.1%)	1 (3.3%)	
T2		9 (10%)	2 (6.7%)	
T3		49 (54%)	20 (67%)	
T4		31 (34%)	7 (23%)	
**Clinical N**	120			0.079
N0		17 (19%)	11 (37%)	
N1		63 (70%)	14 (47%)	
N2		9 (10%)	4 (13%)	
N3		1 (1.1%)	1 (3.3%)	
**Clinical M**	120			
M0		90 (100%)	30 (100%)	
**Clinical Stage**	120			0.7
Stage IIA		2 (2.2%)	0 (0%)	
Stage IIB		19 (21%)	8 (27%)	
Stage IIIA		38 (42%)	15 (50%)	
Stage IIIB		30 (33%)	7 (23%)	
Stage IIIC		1 (1.1%)	0 (0%)	

^1^ n (%); ^2^ Fisher’s exact test; Pearson’s Chi-squared test.

## Data Availability

The raw data supporting the conclusions of this article will be made available by the authors on request.
